# Adverse events signals of enzyme replacement drugs of Gaucher disease: insights from FAERS database analysis

**DOI:** 10.3389/fmed.2026.1726282

**Published:** 2026-04-02

**Authors:** Jiaxun Jiao, Zongyun Li, Lingna Gao, Yuting Wu, Xiaoli Zhu

**Affiliations:** 1 Department of Spinal Surgery, The People's Hospital of Hengshui, Hengshui, Hebei, China; 2 Department of Pharmacy, The People's Hospital of Hengshui, Hengshui, Hebei, China; 3 Department of Pharmacy, The Affiliated Taizhou People's Hospital of Nanjing Medical University, Taizhou, Jiangsu, China

**Keywords:** adverse reaction, disproportionality analysis, enzyme replacement drug, Gaucher disease, signal mining

## Abstract

**Aim:**

This study aims to investigate and compare the post-marketing safety profiles of three widely used enzyme replacement therapies (ERTs) for Gaucher disease—imiglucerase, velaglucerase alfa, and taliglucerase alfa. ERT involves the intravenous administration of recombinant lysosomal enzymes to compensate for the deficiency of β-glucocerebrosidase, thereby reducing the accumulation of glucocerebroside in macrophages and alleviating systemic complications. While ERT has transformed disease management, concerns about long-term adverse events persist. This analysis seeks to identify and characterize distinct safety signals associated with each agent, with a focus on disproportionality analysis rather than establishing causality. The findings aim to inform more vigilant, agent-specific clinical monitoring.

**Methods:**

Adverse event reports were extracted from the FDA Adverse Event Reporting System (FAERS) database. Disproportionality analyses were performed using the Reporting Odds Ratio (ROR), Proportional Reporting Ratio (PRR), Bayesian Confidence Propagation Neural Network (BCPNN), and Multi-item Gamma Poisson Shrinker (MGPS). Patient demographics, significant Preferred Terms (PTs—i.e., specific, medically-encoded adverse event descriptions), and time to onset were examined.

**Results:**

A total of 4,256 AE reports were analyzed. Our signal detection analysis identified a distinct set of statistically significant safety signals for each drug after controlling for background reporting rates. These included 37 significantly associated PTs for imiglucerase, 34 for velaglucerase alfa, and 25 for taliglucerase alfa. The safety profiles differed notably; a significant proportion of the signals for imiglucerase and velaglucerase alfa were infection-related (e.g., respiratory tract infections), whereas such signals were far less prominent for taliglucerase alfa. Unique signals included ear infection for imiglucerase, zinc deficiency for velaglucerase alfa, and hepatic fibrosis for taliglucerase alfa. The median time to onset of these signals differed significantly between agents (*p* < 0.01).

**Conclusion:**

Long-term monitoring is essential during ERT for GD. Clinical vigilance should be heightened for infection-related complications with imiglucerase and velaglucerase alfa, while agent-specific risks like hepatic fibrosis with taliglucerase alfa warrant attention. The observed female predominance in AE reports merits further investigation. These findings are hypothesis-generating; future studies are needed to determine causality.

## Introduction

Gaucher disease (GD) is a rare autosomal recessive lysosomal storage disorder with a global incidence of approximately 1/50,000–1/100,000, while the prevalence in China is lower (1/200,000–1/500,000) (1). This metabolic disorder results from insufficient activity of β-glucocerebrosidase, which is a lysosomal enzyme necessary for the breakdown of glucocerebrosides and their conversion into ceramides and glucose (2). Under normal physiological conditions, GBA facilitates the hydrolysis of glucocerebroside within the lysosomes of macrophages. In GD, the deficient enzyme activity leads to the pathological accumulation of its substrate, glucocerebroside, primarily within the lysosomes of tissue macrophages (Gaucher cells). This accumulation drives the characteristic clinical manifestations, including hepatosplenomegaly, pancytopenia, and skeletal complications such as bone pain and pathological fractures (3). GD exhibits phenotypic heterogeneity, with type II presenting as rapidly progressive neurodegenerative lesions, including ocular movement disorders and brainstem dysfunction, while type III manifests as chronic neurological disorders, such as myoclonus and horizontal eyelid paralysis (4).

Enzyme replacement therapy (ERT) directly addresses this pathophysiology. It involves the periodic intravenous infusion of a recombinant form of the functional enzyme. The administered enzyme is taken up by macrophages, primarily via mannose receptor-mediated endocytosis, and is trafficked to the lysosomes. Here, it compensates for the endogenous deficiency, enabling the catabolism of the accumulated glucocerebroside and thereby alleviating the downstream visceral and hematological pathology (5, 6). Three main ERTs are in clinical use: imiglucerase (approved 1994) (7), velaglucerase alfa (2010) (8), and taliglucerase alfa (2012) (9). While ERT effectively ameliorates visceral and hematological symptoms, concerns regarding long-term safety and the potential for unique adverse event profiles among these agents persist. Spontaneous reporting databases like the FDA Adverse Event Reporting System (FAERS) offer a valuable resource for post-marketing surveillance to detect potential safety signals that may not be fully apparent in pre-approval clinical trials (10, 11).

This study aims to conduct a comprehensive pharmacovigilance analysis of adverse events associated with imiglucerase, velaglucerase alfa, and taliglucerase alfa using the FAERS database. We will employ multiple disproportionality analysis algorithms to identify statistically significant safety signals (referred to as Preferred Term or PT signals) for each drug. Furthermore, we will analyze patient demographics and temporal patterns, specifically the time from therapy initiation to adverse event onset. The primary objective is to detect and characterize potential differences in the safety profiles of these three ERTs, thereby generating hypotheses for targeted monitoring and future research. It is crucial to emphasize that this analysis identifies statistical associations and does not establish causality.

## Materials and methods

### Data sources

The data for this study were sourced from the FAERS database. The basic characteristics of the three enzyme replacement therapies (ERTs) for Gaucher disease (GD) are summarized in Table 1. At the time of analysis, the FDA had only released data up to the third quarter of 2024. All ASCII data packages for the three drugs were extracted, covering the period from the second quarter of 2012 to the third quarter of 2024.

**Table 1:** Basic information of the three enzyme replacement therapies for Gaucher disease.

Generic names	Product names	Time to market
Imiglucerase	Cerezyme	1994.05
Velaglucerase alfa	Vpriv	2010.02
Taliglucerase alfa	Elelyso	2012.05

Data cleaning was performed according to the FDA’s standard procedure for FAERS data management. First, deduplication was conducted by identifying reports sharing identical CASEID values and retaining the report with the most recent FDA_DT. If multiple reports shared the same CASEID and FDA_DT, the report with the highest PRIMARYID was selected. Subsequently, reports for the three target ERTs were identified by searching for generic names and brand names in the drug name field. Specifically, we included the following search terms to ensure comprehensiveness:

Imiglucerase: ‘imiglucerase’ and its brand name ‘Cerezyme’.

Velaglucerase alfa: ‘velaglucerase’ and its brand names ‘VPRIV’, ‘Vpriv’.

Taliglucerase alfa: ‘taliglucerase’ and its brand names ‘Elelyso’, ‘Uplyso’.

Only reports listing these drugs as the primary suspect (PS) agents were retained for further analysis. Uninterpretable AEs were excluded. The identified AEs were categorized according to the System Organ Class (SOC) and Preferred Term (PT) classifications within the Medical Dictionary for Regulatory Activities (MedDRA) terminology. AE signals that could not be determined were excluded.

To evaluate the AEs, we employed disproportionality analysis: Reporting Odds Ratio (ROR), Proportional Reporting Ratio (PRR), Bayesian Confidence Propagation Neural Network (BCPNN) and Multiitem Gamma Poisson Shrinker (MGPS). These are common algorithms for disproportionality analysis and are currently widely used by the World Health Organization and the FDA (12). ROR is the ratio of the odds of reporting a specific adverse event for a drug compared to the odds of reporting the same event for all other drugs in a database (13). PRR compares the proportion of reports of a specific adverse event for a drug to the proportion of the same event for all other drugs (14). BCPNN is a Bayesian statistical approach that estimates the probability of association between a drug and an adverse event by considering prior distributions and observed data (15). MGPS is a Bayesian data mining method that shrinks observed disproportionality estimates toward the mean using a gamma-Poisson model, reducing noise in small sample sizes (16). The calculation formulas and positive safety signal thresholds were provided in Tables 2, 3. If four criteria are met, the result is considered a potential signal. Given the exploratory nature of pharmacovigilance signal detection, statistical significance does not imply clinical causality. For signals with exceptionally high association strength (e.g., ROR ≥ 10) or significant clinical implications, we provided brief clinical interpretations in the main text, considering potential mechanisms or confounding factors. All analyses were performed using R software version 4.3.2 (R Foundation for Statistical Computing, Vienna, Austria).

**Table 2:** Fourfold table of measures of disproportionality.

Drugs	Target AEs	Other AEs	Total
Target drugs	a	b	a + b
Other drugs	c	d	c + d
Total	a + c	b + d	a + b + c + d

**Table 3:** Four major algorithms used for signal detection.

Algorithms	Equation	Signal detection criteria
ROR	ROR = (a/c)/(b/d)	95% CI > 1, *N* ≥ 3
	95%CI = e^ln(ROR) ± 1.96(1/a + 1/b + 1/c + 1/d)^0.5^	
PRR	PRR = [a(c + d)]/[c/(a + b)]	PRR ≥ 2, *χ*^2^ ≥ 4, *N* ≥ 3
	χ^2^ = [(ad-bc)^2](a + b + c + d)/[(a + b)(c + d)(a + c)(b + d)]	
BCPNN	IC = log_2_a(a + b + c + d)/(a + c)/(a + b)	IC025 > 0
	95%CI = E(IC) ± 2 V(IC)^0.5	
MGPS	EBGM = a(a + b + c + d)/(a + c)/(a + b)	EBGM05 > 2
	95%CI = e^ln(EBGM) ± 1.96(1/a + 1/b + 1/c + 1/d)^0.5^	

“IC025” the lower limit of 95% CI of the IC, “EBGM05” the lower limit of 95% CI of EBGM.

## Results

### Population characteristics

From the second quarter of 2012 to the third quarter of 2024, total 4,256 AE reports were analyzed through disproportionality analysis, including 2,251 for imiglucerase, 1,600 for velaglucerase alfa and 405 for taliglucerase alfa. Female patients accounted for 52.7% cases, and males accounted for 36.8%. A higher proportion of AEs were reported in females. Patients aged between 18 and 65 years accounted for a higher proportion (37.0%) than patients under 18 years old (17.4%) and patients over 65 years old (13.0%). The US (56.9%) reported the largest number of AEs (Table 4).

**Table 4:** Cases characteristics of in the FAERS database.

Characteristic	Imiglucerase (*n* = 2,251)		Velaglucerase alfa (*n* = 1,600)		Taliglucerase alfa (*n* = 405)		Total (*n* = 4,256)	
	Case (*n*)	Proportion (%)	Case (*n*)	Proportion (%)	Case (*n*)	Proportion (%)	Case (*n*)	Proportion (%)
Sex								
Male	781	34.7	620	38.8	165	40.7	1,566	36.8
Female	1,246	55.4	786	49.1	209	51.6	2,241	52.7
Missing	224	10.0	194	12.1	31	7.7	449	10.5
Age								
<18 years	507	22.5	183	11.4	50	12.3	740	17.4
18–65 years	857	38.1	514	32.1	204	50.4	1,575	37.0
>65 years	241	10.7	239	14.9	75	18.5	555	13.0
Missing	644	28.6	664	41.5	76	18.8	1,384	32.5
Reporting country (Top 3)	US 1529	67.9	US 720	45.0	US 171	42.2	US 2420	56.9
	EG 76	3.4	ISR 336	21.0	BR 99	24.4	ISR 402	9.4
	UK 55	2.4	UK 97	6.1	ISR 66	16.3	UK 152	3.6

US, United States; EG, Egypt; ISR, Israel; UK, United Kingdom; BR, Brazil.

### Signal detection

**Figure 1:**
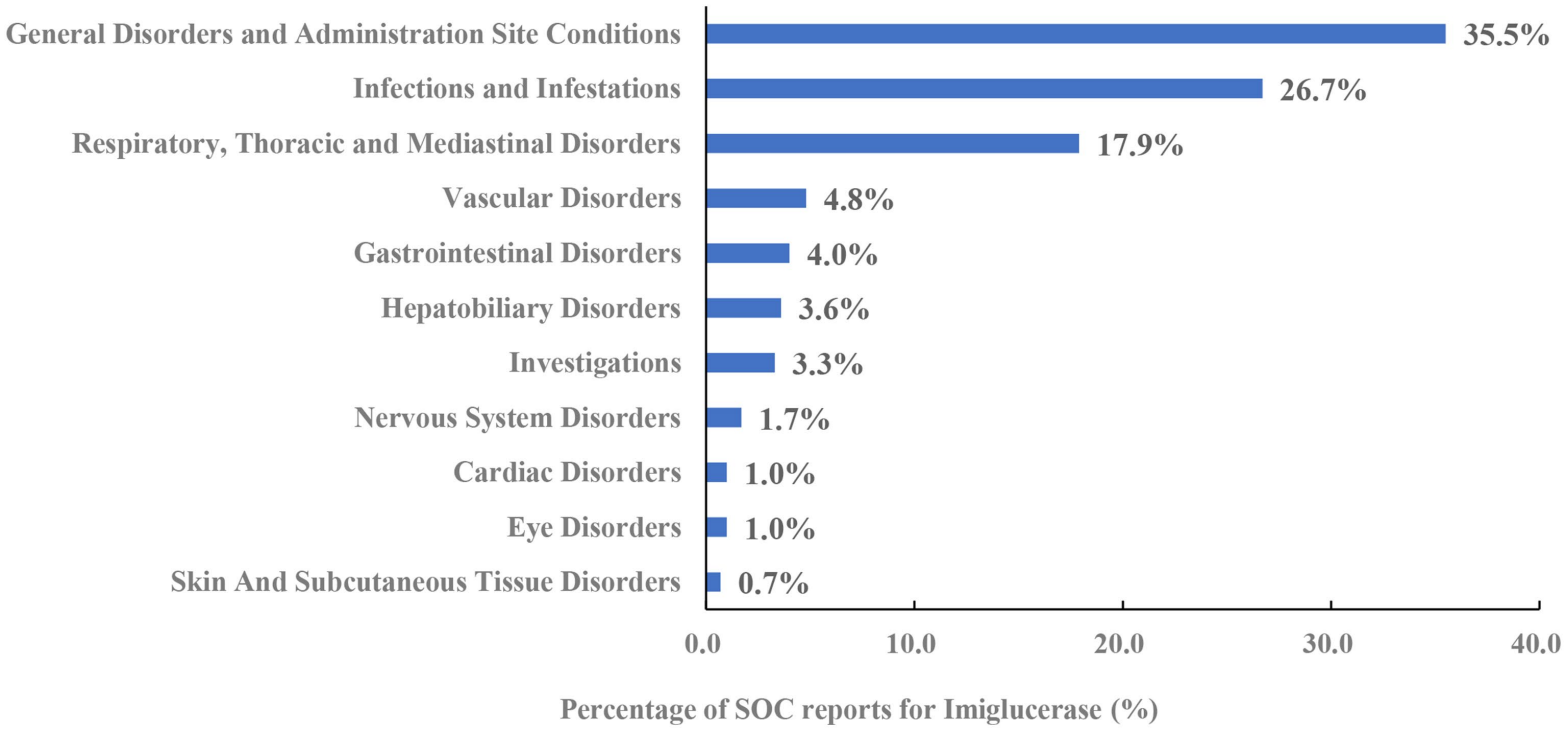
System organ class distribution of positively associated preferred terms for imiglucerase.

**Figure 2:**
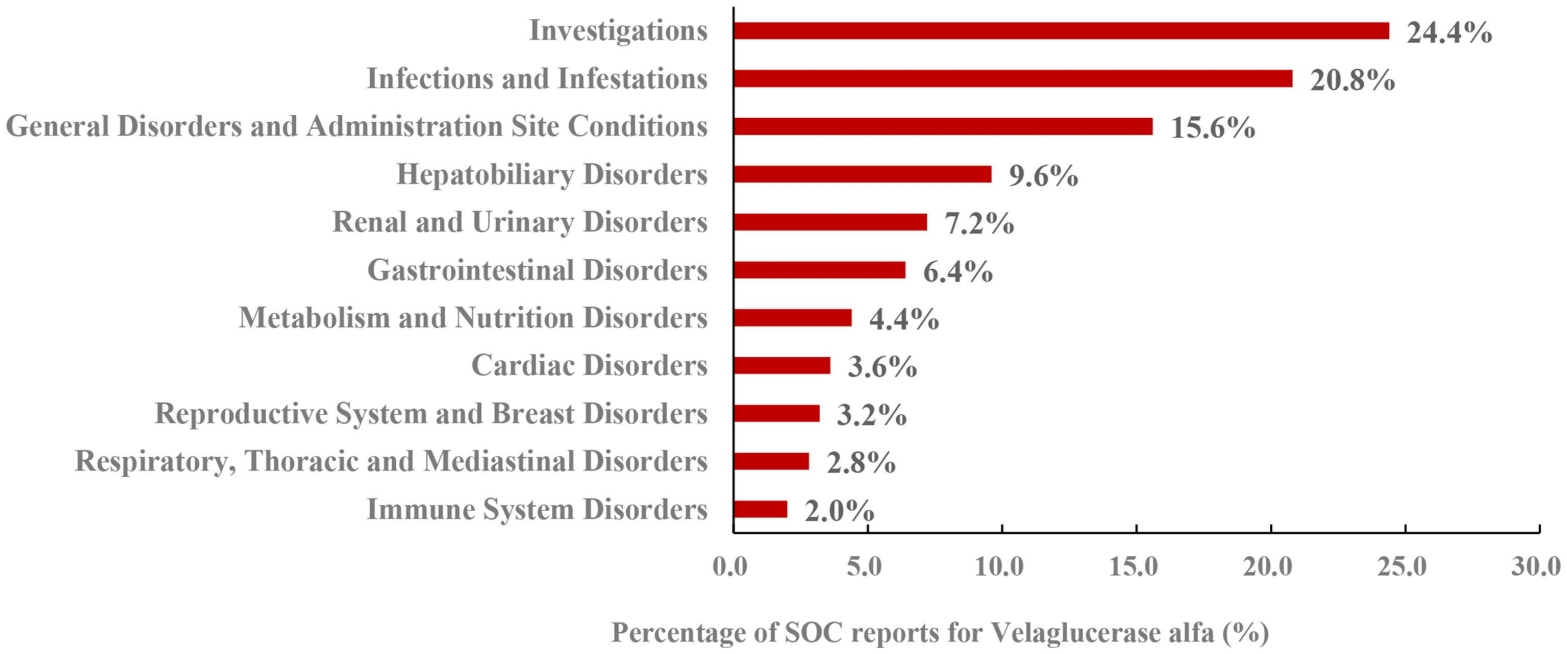
System organ class distribution of positively associated preferred terms for velaglucerase alfa.

**Figure 3:**
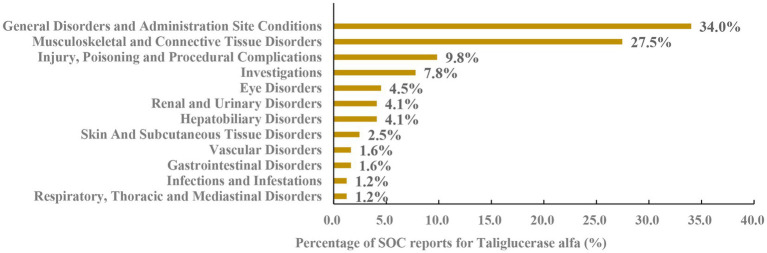
System organ class distribution of positively associated preferred terms for taliglucerase alfa.

Imiglucerase, velaglucerase alfa and taliglucerase alfa yielded 1,342, 1,367 and 730 PT signals, respectively. Following application of four algorithmic criteria, manual screening was performed to exclude signals attributable to disease progression or drug-unrelated adverse reactions. This yielded 37, 34 and 25 validated positive signals for each respective agent. The distribution of these signals across System Organ Classes (SOCs) is illustrated in Figures 1–3, revealing distinct safety profiles.

Among the positive PTs associated with imiglucerase, 11 SOCs were involved, with the top three being: Respiratory, Thoracic and Mediastinal Disorders; Infections and Infestations; and General Disorders and Administration Site Conditions (Figure 1). This SOC distribution indicates a notable burden of respiratory complications and infections with imiglucerase, consistent with its known profile, alongside expected administration site reactions.

Velaglucerase alfa was also associated with 11 SOCs, with the top three being: Investigations; Infections and Infestations; and General Disorders and Administration Site Conditions (Figure 2). The prominence of the Investigations SOC, which includes laboratory abnormalities such as zinc deficiency, points to potential metabolic or biochemical effects that require clinical monitoring, in addition to its association with infections.

Taliglucerase alfa was associated with 12 SOCs, with the top three being: General Disorders and Administration Site Conditions; Musculoskeletal and Connective Tissue Disorders; and Injury, Poisoning and Procedural Complications (Figure 3). This pattern suggests a safety profile where musculoskeletal complaints and pain-related conditions are more prominent relative to the other agents, alongside common infusion-related reactions.

All three therapeutic agents (imiglucerase, velaglucerase alfa and taliglucerase alfa) demonstrated AEs profiles across multiple SOCs. Notable adverse reactions within the General Disorders and Administration Site Conditions SOC included pyrexia, infusion site pain, swelling, and exudation. Hypersensitivity reactions manifested through symptoms such as chest discomfort, dyspnea and cyanosis. Cholelithiasis, nephrolithiasis and retinal detachment emerged as positively reported PTs common to all three therapeutic agents. Infection dominated the safety profiles of imiglucerase and velaglucerase alfa, whereas taliglucerase alfa showed minimal infectious complications. Imiglucerase exhibited unique positively PTs, including ear infection, while velaglucerase alfa demonstrated distinct PTs such as zinc deficiency, and taliglucerase alfa showed specific PTs of hepatic fibrosis (Table 5).

**Table 5:** ADR signals of the top 15 reported cases: the “Cases” column indicates the number of unique reports after deduplication^1^.

Drugs	Pts	Cases	ROR (CI95%)	PRR (*Χ*^2^)	EBGM (EBGM05)	IC (IC025)
Imiglucerase	Pyrexia	125	3.8 (3.18–4.53)	3.74 (252.02)	3.74 (3.22)	1.9 (1.64)
	Lower Respiratory Tract Infection	28	5.73 (3.96–8.31)	5.71 (108.87)	5.71 (4.18)	2.51 (1.98)
	Respiratory Failure	27	4.15 (2.84–6.06)	4.14 (64.22)	4.13 (3.01)	2.05 (1.5)
	Upper Respiratory Tract Infection	17	3.63 (2.25–5.84)	3.62 (32.26)	3.62 (2.43)	1.86 (1.17)
	Hematemesis	14	6.08 (3.60–10.28)	6.07 (59.25)	6.06 (3.91)	2.6 (1.85)
	Ear Infection	14	5.03 (2.98–8.5)	5.02 (45.07)	5.02 (3.24)	2.33 (1.58)
	Body Temperature Increased	11	5.83 (3.23–10.54)	5.82 (43.92)	5.82 (3.55)	2.54 (1.71)
	Pneumonia Aspiration	11	4.53 (2.51–8.19)	4.52 (30.18)	4.52 (2.76)	2.18 (1.34)
	Respiratory Distress	11	4.50 (2.49–8.14)	4.5 (29.92)	4.50(2.74)	2.17 (1.33)
	Infusion Site Extravasation	10	13.82 (7.43–25.71)	13.8 (118.44)	13.77 (8.19)	3.78 (2.91)
	Cyanosis	10	8.17 (4.39–15.19)	8.15 (62.7)	8.15 (4.85)	3.03 (2.15)
	Respiratory Tract Infection	10	3.72 (2.00–6.91)	3.71 (19.81)	3.71 (2.21)	1.89 (1.02)
	Infusion Site Pain	9	6.17 (3.21–11.87)	6.17 (38.93)	6.16 (3.56)	2.62 (1.71)
	Cholelithiasis	9	3.68 (1.91–7.08)	3.68 (17.55)	3.68 (2.13)	1.88 (0.97)
	Viral Upper Respiratory Tract Infection	8	12.94 (6.46–25.90)	12.92 (87.83)	12.9 (7.22)	3.69 (2.73)
	Pulmonary Hypertension	8	5.59 (2.67–11.74)	5.59 (26.36)	5.59 (3.00)	2.48 (1.46)
	Bronchospasm	7	4.57 (2.18–9.6)	4.57 (19.49)	4.56 (2.45)	2.19 (1.17)
	Hypokinesia	7	3.8 (1.81–7.97)	3.8 (14.41)	3.79 (2.04)	1.92 (0.90)
	Gastroenteritis Viral	7	21.98 (9.86–49)	21.96 (119.6)	21.88 (11.19)	4.45 (3.36)
	Portal Hypertension	6	5.36 (2.41–11.95)	5.36 (21.27)	5.36 (2.74)	2.42 (1.33)
	Pharyngitis Streptococcal	6	4.73 (2.12–10.53)	4.72 (17.6)	4.72 (2.42)	2.24 (1.15)
	Tachypnoea	6	4.16 (1.87–9.28)	4.16 (14.41)	4.16 (2.13)	2.06 (0.96)
	Circulatory Collapse	6	7.75 (3.22–18.65)	7.75 (29.36)	7.74 (3.71)	2.95 (1.77)
	Apnoea	5	7.4 (3.08–17.8)	7.4 (27.63)	7.39 (3.55)	2.89 (1.71)
	Infusion Site Swelling	5	18.26 (6.84–48.72)	18.24 (65.02)	18.2 (8.00)	4.19 (2.89)
	Vein Rupture	4	16.58 (6.21–44.24)	16.57 (58.37)	16.53 (7.27)	4.05 (2.75)
	Stridor	4	12.63 (4.73–33.7)	12.62 (42.73)	12.6 (5.54)	3.66 (2.36)
	Cardiopulmonary Failure	4	11.79 (4.42–31.45)	11.78 (39.40)	11.76 (5.18)	3.56 (2.26)
	Bronchiolitis	4	7.29 (2.73–19.43)	7.28 (21.66)	7.28 (3.20)	2.86 (1.57)
	Tonsillitis	4	5.77 (2.16–15.39)	5.77 (15.75)	5.76 (2.54)	2.53 (1.24)
	Pulmonary Hemorrhage	4	4.69 (1.76–12.49)	4.68 (11.58)	4.68 (2.06)	2.23 (0.94)
	Retinal Detachment	4	20.56 (6.62–63.89)	20.55 (55.63)	20.49 (7.94)	4.36 (2.91)
	Antibody Test Positive	3	12.6 (4.06–39.12)	12.6 (31.96)	12.57 (4.87)	3.65 (2.21)
	Ingrowing Nail	3	11.28 (3.63–35.02)	11.28 (28.05)	11.26 (4.36)	3.49 (2.05)
	Neonatal Respiratory Distress Syndrome	3	10.85 (3.49–33.68)	10.84 (26.76)	10.83 (4.2)	3.44 (1.99)
	Varices Oesophageal	3	7.15 (2.3–22.18)	7.14 (15.83)	7.14 (2.77)	2.84 (1.39)
	Rhinovirus Infection	3	5.59 (2.67–11.74)	5.59 (26.36)	5.59 (3.00)	2.48 (1.46)
Velaglucerase Alfa	Blood Pressure Increased	45	2.88 (2.15–3.86)	2.87 (54.87)	2.87 (2.24)	1.52 (1.09)
	Cholelithiasis	19	7.64 (4.87–11.98)	7.62 (109.11)	7.61 (5.22)	2.93 (2.28)
	Cellulitis	17	3.34 (2.08–5.38)	3.33 (27.79)	3.33 (2.24)	1.74 (1.06)
	Nephrolithiasis	15	3.2 (1.93–5.31)	3.2 (22.64)	3.19 (2.09)	1.68 (0.95)
	Infusion Site Pain	12	8.07 (4.58–14.23)	8.06 (74.13)	8.05 (5.01)	3.01 (2.21)
	Ascites	10	5.19 (2.79–9.66)	5.19 (33.79)	5.18 (3.08)	2.37 (1.50)
	Body Temperature Increased	10	3.52 (1.89–6.55)	3.52 (18.01)	3.52 (2.09)	1.81 (0.94)
	Angina Pectoris	9	3.64 (1.89–7.00)	3.64 (17.22)	3.64 (2.10)	1.86 (0.95)
	Infusion Site Erythema	7	7.74 (3.69–16.24)	7.73 (40.97)	7.72 (4.15)	2.95 (1.93)
	Infusion Site Extravasation	6	8.11 (3.64–18.07)	8.1 (37.33)	8.1 (4.14)	3.02 (1.93)
	Zinc Deficiency	5	254.8 (104.19–623.11)	254.6 (1214.45)	244.85 (115.86)	7.94 (6.73)
	Respiratory Syncytial Virus Infection	5	11.35 (4.72–27.29)	11.34 (47.05)	11.32 (5.43)	3.5 (2.32)
	Multiple Allergies	5	8.93 (3.71–21.48)	8.92 (35.14)	8.91 (4.28)	3.16 (1.98)
	Cholecystitis Acute	5	5.9 (2.45–14.18)	5.9 (20.31)	5.89 (2.83)	2.56 (1.38)
	Appendicitis	5	5.89 (2.45–14.17)	5.89 (20.27)	5.88 (2.82)	2.56 (1.38)
	Pharyngitis Streptococcal	5	4.38 (1.82–10.53)	4.38 (13.02)	4.37 (2.10)	2.13 (0.95)
	Ovarian Cyst	5	5.49 (2.28–13.21)	5.49 (18.34)	5.49 (2.63)	2.46 (1.28)
	Pyelonephritis	5	5.89 (2.45–14.17)	5.89 (20.27)	5.88 (2.82)	2.56 (1.38)
	Zinc Deficiency	5	254.8 (104.19–623.11)	254.6 (1214.45)	244.85 (115.86)	7.94 (6.73)
	Rhinitis	4	4.87 (1.83–12.98)	4.87 (12.28)	4.86 (2.14)	2.28 (0.99)
	Upper Respiratory Tract Inflammation	4	33.88 (12.68–90.53)	33.86 (126.87)	33.68 (14.80)	5.07 (3.78)
	Infusion Site Rash	4	27.69 (10.37–73.96)	27.67 (102.4)	27.56 (12.11)	4.78 (3.49)
	Infusion Site Swelling	4	5.8 (2.18–15.47)	5.8 (15.86)	5.79 (2.55)	2.53 (1.24)
	Rhinovirus Infection	3	7 (2.26–21.73)	7 (15.41)	6.99 (2.71)	2.81 (1.36)
	Hypernatraemia	3	6.46 (2.08–20.05)	6.46 (13.82)	6.45 (2.50)	2.69 (1.25)
	Rhinitis Allergic	3	7.26 (2.34–22.52)	7.25 (16.16)	7.25 (2.81)	2.86 (1.41)
	Pneumonia Klebsiella	3	16.38 (5.27–50.89)	16.38 (43.20)	16.34 (6.33)	4.03 (2.58)
	Benign Prostatic Hyperplasia	3	5.96 (1.92–18.5)	5.96 (12.37)	5.96 (2.31)	2.57 (1.13)
	Colitis Ischaemic	3	5.26 (1.69–16.32)	5.26 (10.33)	5.25 (2.04)	2.39 (0.95)
	Ureterolithiasis	3	21.96 (7.07–68.24)	21.95 (59.78)	21.88 (8.47)	4.45 (3.00)
	Infusion Site Hemorrhage	3	7.94 (2.56–24.64)	7.94 (18.17)	7.93 (3.07)	2.99 (1.54)
	Infusion Site Bruising	3	13.32 (4.29–41.37)	13.32 (34.10)	13.29 (5.15)	3.73 (2.29)
	Haemorrhoidal Hemorrhage	3	5.72 (1.84–17.75)	5.72 (11.67)	5.71 (2.21)	2.51 (1.07)
	Neutralizing Antibodies Positive	3	106.73 (34.09–334.15)	106.68 (308.88)	104.93 (40.38)	6.71 (5.25)
Taliglucerase Alfa	Pyrexia	36	2.83 (2.04–3.94)	2.81 (42.09)	2.81 (2.13)	1.49 (1.01)
	Pain In Extremity	34	2.98 (2.12–4.18)	2.95 (44.12)	2.95 (2.22)	1.56 (1.07)
	Back Pain	30	3.35 (2.33–4.80)	3.32 (48.72)	3.32 (2.45)	1.73 (1.21)
	Infusion Related Reaction	24	9.44 (6.31–14.11)	9.35 (179.14)	9.35 (6.68)	3.22 (2.64)
	Chest Pain	22	3.53 (2.32–5.38)	3.51 (39.59)	3.51 (2.47)	1.81 (1.21)
	Chest Discomfort	17	4.50(2.79–7.26)	4.48 (45.99)	4.48 (3.00)	2.16 (1.48)
	Heart Rate Decreased	9	6.36 (3.31–12.24)	6.34 (40.50)	6.34 (3.67)	2.66 (1.75)
	Nephrolithiasis	7	3.98 (1.89–8.35)	3.97 (15.54)	3.97 (2.13)	1.99 (0.97)
	Cholelithiasis	6	18.37 (8.24–40.94)	18.32 (98.17)	18.3 (9.36)	4.19 (3.10)
	Retinal Detachment	6	6.41 (2.88–14.28)	6.39 (27.30)	6.39 (3.27)	2.68 (1.58)
	Lacrimation Increased	5	4.23 (1.76–10.18)	4.23 (12.32)	4.23 (2.03)	2.08 (0.90)
	Cyanosis	4	36.38 (13.63–97.13)	36.32 (137.12)	36.25 (15.94)	5.18 (3.89)
	Hepatic Fibrosis	4	14.39 (5.40–38.40)	14.37 (49.73)	14.36 (6.32)	3.84 (2.55)
	Blood Pressure Systolic Increased	4	8.51 (3.19–22.70)	8.5 (26.46)	8.5 (3.74)	3.09 (1.79)
	Infusion Site Extravasation	4	7.15 (2.68–19.08)	7.14 (21.13)	7.14 (3.14)	2.84 (1.54)
	Infusion Site Pain	4	7.15 (2.68–19.08)	7.14 (21.13)	7.14 (3.14)	2.84 (1.54)
	Lipoedema	3	17.61 (5.67–54.69)	17.59 (46.91)	17.58 (6.81)	4.14 (2.69)
	Anal Fissure	3	14.44 (4.65–44.84)	14.43 (37.46)	14.41 (5.59)	3.85 (2.4)
	Tachypnoea	3	6.16 (1.99–19.12)	6.16 (12.95)	6.15 (2.39)	2.62 (1.18)
	Tendonitis	3	5.52 (1.78–17.14)	5.52 (11.10)	5.52 (2.14)	2.46 (1.02)
	Antibody Test Positive	3	53.66 (17.26–166.81)	53.6 (154.36)	53.43 (20.69)	5.74 (4.29)
	Paresthesia Oral	3	5.61 (1.81–17.4)	5.6 (11.34)	5.6 (2.17)	2.49 (1.04)
	Renal Pain	3	7.45 (2.40–23.12)	7.44 (16.72)	7.44 (2.88)	2.9 (1.45)
	Body Temperature Decreased	3	7.24 (2.33–22.46)	7.23 (16.09)	7.22 (2.80)	2.85 (1.41)
	Gastroenteritis	3	5.92 (1.91–18.36)	5.91 (12.24)	5.91 (2.29)	2.56 (1.12)

^1^The number of unique reports for each Preferred Term (PT) after removing duplicate entries.

Among the unique agent-specific signals, the extraordinarily high ROR for zinc deficiency associated with velaglucerase alfa (ROR = 254.8, Table 5) warrants attention. Although based on a small number of cases (*n* = 5), this strong statistical signal may suggest a potential association requiring further investigation. The underlying mechanism is unclear but could hypothetically relate to altered zinc homeostasis or reporting bias.

The pharmacovigilance analysis revealed distinct safety signal profiles among the three agents. Imiglucerase demonstrated the strongest association with five adverse events: pyrexia, lower respiratory tract infection, respiratory failure, upper respiratory tract infection and hematemesis. Velaglucerase alfa demonstrated predominant signals including blood pressure increased, cholelithiasis, cellulitis, nephrolithiasis and infusion site pain. Taliglucerase alfa showed characteristic signals of pyrexia, pain in extremity, back pain, infusion related reaction and chest pain (Table 5).

Furthermore, several signals with notable ROR values were observed. For imiglucerase, infusion site extravasation (ROR = 13.82) and viral upper respiratory tract infection (ROR = 12.94) showed strong associations. For velaglucerase alfa, besides zinc deficiency, infusion site reactions (pain, erythema, and extravasation) and respiratory syncytial virus infection displayed elevated RORs. The signal for hepatic fibrosis associated with taliglucerase alfa (ROR = 14.39, though not in the top 15 by case count) is clinically significant given the hepatic involvement in GD, necessitating differentiation between drug effect and natural disease progression.

### Onset time of events

**Figure 4:**
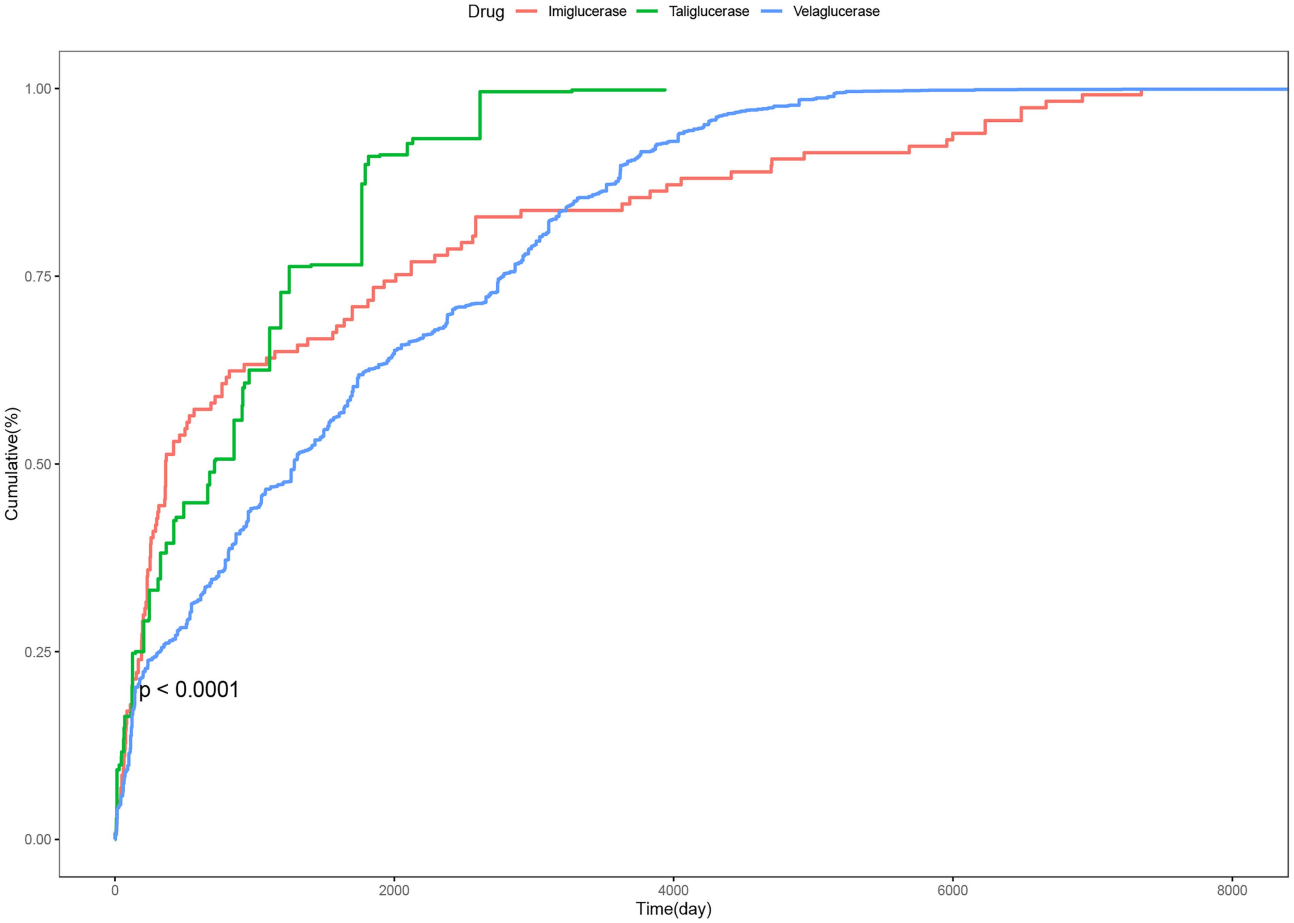
Time cumulative distribution curve for AEs.

The median onset time was 363 days (IQR191-2011 days) for imiglucerase1284 days (IQR324-2781 days) for velaglucerase alfa and 713 days (IQR 191–1,248 days) for taliglucerase alfa. Significant differences in cumulative incidence rates of AEs were observed among the three therapeutic agents (*p* < 0.0001) (Figure 4).

### Incidence of adverse events

There were 37 cases of imiglucerase positive PT, with a total of 420 reports. Among them, 229 were serious adverse events, mainly distributed as follows: Pyrexia (68), Respiratory Failure (26), Lower Respiratory Tract Infection (14), Hematemesis (12), Pneumonia Aspiration (11).

There were 34 cases of velaglucerase alfa positive PT, with a total of 244 reports. Among them, 143 were serious adverse events, mainly distributed as follows: Increased Blood Pressure (27), Cholelithiasis (12), Cellulitis (11), Ascites (10), Nephrolithiasis (8), Increased Body Temperature (8).

There were 25 cases of taliglucerase alfa positive PT, with a total of 243 reports. Among them, 76 were serious adverse events, mainly distributed as follows: Pyrexia (13), Pain in Limbs (13), Chest Pain (11), Back Pain (7), Nephrolithiasis (5). The details were shown in Table 6.

**Table 6:** Incidence of adverse events.

Drugs	Pts	Serious adverse events
Imiglucerase	Pyrexia	68
	Respiratory Failure	26
	Lower Respiratory Tract Infection	14
	Hematemesis	12
	Pneumonia Aspiration	11
	Respiratory Distress	9
	Cyanosis	9
	Circulatory Collapse	6
	Cholelithiasis	5
	Bronchospasm	5
	Hypokinesia	5
	Tachypnoea	5
	Upper Respiratory Tract Infection	4
	Portal Hypertension	4
	Stridor	4
	Cardiopulmonary Failure	4
	Pulmonary Hemorrhage	4
	Ear Infection	3
	Body Temperature Increased	3
	Respiratory Tract Infection	3
	Apnoea	3
	Bronchiolitis	3
	Neonatal Respiratory Distress Syndrome	3
	Varices Oesophageal	3
	Rhinovirus Infection	3
	Infusion Site Extravasation	2
	Pulmonary Hypertension	2
	Antibody Test Positive	2
	Viral Upper Respiratory Tract Infection	1
	Gastroenteritis Viral	1
	Pharyngitis Streptococcal	1
	Vein Rupture	1
	Infusion Site Pain	0
	Infusion Site Swelling	0
	Tonsillitis	0
	Retinal Detachment	0
	Ingrowing Nail	0
Velaglucerase Alfa	Blood Pressure Increased	27
	Cholelithiasis	12
	Cellulitis	11
	Ascites	10
	Nephrolithiasis	8
	Body Temperature Increased	8
	Respiratory Syncytial Virus Infection	5
	Cholecystitis Acute	5
	Appendicitis	5
	Infusion Site Pain	4
	Pyelonephritis	4
	Infusion Site Erythema	3
	Multiple Allergies	3
	Pharyngitis Streptococcal	3
	Ovarian Cyst	3
	Rhinovirus Infection	3
	Hypernatraemia	3
	Pneumonia Klebsiella	3
	Ureterolithiasis	3
	Haemorrhoidal Hemorrhage	3
	Angina Pectoris	2
	Infusion Site Extravasation	2
	Infusion Site Swelling	2
	Benign Prostatic Hyperplasia	2
	Colitis Ischaemic	2
	Infusion Site Hemorrhage	2
	Zinc Deficiency	1
	Zinc Deficiency	1
	Rhinitis	1
	Upper Respiratory Tract Inflammation	1
	Infusion Site Bruising	1
	Infusion Site Rash	0
	Rhinitis Allergic	0
	Neutralizing Antibodies Positive	0
Taliglucerase Alfa	Pyrexia	13
	Pain In Extremity	13
	Chest Pain	11
	Back Pain	7
	Nephrolithiasis	5
	Chest Discomfort	4
	Infusion Related Reaction	3
	Gastroenteritis	3
	Heart Rate Decreased	2
	Cholelithiasis	2
	Retinal Detachment	2
	Lacrimation Increased	2
	Anal Fissure	2
	Paresthesia Oral	2
	Cyanosis	1
	Hepatic Fibrosis	1
	Infusion Site Extravasation	1
	Infusion Site Pain	1
	Tendonitis	1
	Blood Pressure Systolic Increased	0
	Lipoedema	0
	Tachypnoea	0
	Antibody Test Positive	0
	Renal Pain	0
	Body Temperature Decreased	0

## Discussion

### Summary of main findings

This study analyzed real-world safety data for the three primary ERTs used in GD: imiglucerase, velaglucerase alfa, and taliglucerase alfa. The key findings are: (1) More side effects were reported by women (52.7%) than by men (36.8%). The majority of reports came from adults aged 18–65. (2) Imiglucerase and velaglucerase alfa were strongly linked to reports of infections, like respiratory illnesses. This was less common with taliglucerase alfa. Each drug also showed unique signals: ear infections for imiglucerase, a very strong signal for zinc deficiency for velaglucerase alfa, and liver fibrosis for taliglucerase alfa. (3) All three drugs were associated with reports of gallstones, kidney stones, and retinal detachment, suggesting these could be potential class effects. (4) Side effects often started long after beginning treatment (median times ranging from about 1 to 3.5 years), highlighting the need for long-term patient monitoring.

### Interpretation of safety signals and clinical implications

Infection risk: The higher reporting of infections with imiglucerase and velaglucerase alfa may be related to their production in mammalian cells, which can sometimes trigger the immune system differently than plant-derived taliglucerase alfa (17, 18). This finding suggests doctors should be particularly watchful for signs of infection in patients on the former two drugs.

Drug-specific signals: The unique signals require different clinical considerations. The zinc deficiency signal for velaglucerase alfa, while very strong statistically, is based on few cases and needs further study; checking zinc levels in symptomatic patients may be prudent. The liver fibrosis signal for taliglucerase alfa is complex because liver problems can also be part of GD itself, so careful monitoring is needed to tell the difference. The ear infection signal for imiglucerase suggests patients with ear complaints might benefit from an ear examination.

Common signals (Class effects): The association of all ERTs with gallstones, kidney stones, and eye problems indicates that regular check-ups for these conditions should be part of the care plan for any GD patient on long-term ERT, regardless of which specific drug they use.

Time to onset: The delayed onset of many side effects means that safety monitoring must continue for years, well beyond the typical duration of clinical trials.

### Exploration of gender and age disparities

The higher rate of adverse event reports among women is a notable finding. While GD affects men and women equally, several factors could explain this disparity. Biologically, differences in immune system function or hormone levels between sexes might influence how individuals react to or report side effects (19). Behaviorally, women may be more likely to report health issues or have more frequent contact with healthcare providers. Furthermore, because GD can specifically impact women’s reproductive health (e.g., causing heavy periods), more women of childbearing age may be on treatment, increasing the overall number of reports from this group (20). The concentration of reports in the 18–65 age group likely reflects the main population receiving these lifelong therapies. Although this does not prove the drugs are less safe for women, it underscores the importance of including gender as a factor in safety monitoring and future research.

### Limitations

This study uses voluntary reports, which means we cannot calculate exactly how often side effects occur. Reports can be influenced by many factors (e.g., increased reporting for newer drugs), and we cannot definitively prove that the drug caused the event, especially when the event (like liver fibrosis) could also be due to the underlying disease. The findings, especially from small numbers of reports, should be seen as generating hypotheses for further research.

### Future perspectives

This study points to several important areas for future work: (1) Confirmation: The potential risks identified here, especially the unique ones like zinc deficiency, need to be confirmed in dedicated prospective studies that can better determine cause and effect. (2) Mechanistic research: Studies are needed to understand why the infection risk might differ between drugs, perhaps by exploring how their different manufacturing processes affect the immune system. (3) Personalized monitoring: Future research should try to identify which patients are at highest risk for specific side effects based on their GD type, genetics, or other factors, allowing for more tailored monitoring plans.

## Conclusion

This analysis reveals distinct safety patterns among the three ERTs for Gaucher disease. It suggests that treatment decisions and long-term monitoring strategies could be optimized by considering the specific drug used, with particular attention to infection risk for imiglucerase/velaglucerase alfa, unique metabolic or organ-specific signals, and potential class-wide effects (summarized in Table 7). The findings also highlight the need for sustained, long-term vigilance in all treated patients.

**Table 7:** Proposed drug-specific risk-monitoring recommendations based on pharmacovigilance signals.

Drug	Key safety signals from study	Proposed monitoring recommendations
Imiglucerase	Heightened infection risk (respiratory, ear infection)	-Vigilance for signs/symptoms of respiratory and ear infections. -Consider periodic ear examination in symptomatic patients. -Standard monitoring for infusion-related reactions and hypersensitivity.
Velaglucerase alfa	Hypertension, zinc deficiency, infection risk	-Pre- and intra-infusion blood pressure monitoring. -Consider serum zinc level assessment in patients with suggestive clinical features (e.g., dysgeusia, dermatitis). -Surveillance for infections.
Taliglucerase alfa	Hepatic fibrosis signal, infusion-related reactions	-Regular liver function tests (LFTs). -Consider periodic non-invasive liver fibrosis assessment (e.g., transient elastography) to help differentiate drug effect from GD progression, especially in patients with baseline liver involvement or rising LFTs. -Standard monitoring for infusion-related reactions.
All Three ERTs	Crystalloid pathologies (cholelithiasis, nephrolithiasis), retinal complications, delayed-onset AEs	-Consider baseline and periodic abdominal imaging (ultrasound) based on clinical judgment for lithiasis. -Recommend regular ophthalmological assessments. -Emphasize long-term (>1 year) surveillance for all patients, as AEs can manifest years after treatment initiation.

These recommendations are based on signal detection from a spontaneous reporting database and are intended to guide enhanced clinical vigilance and hypothesis generation. They do not establish causality and should be implemented considering individual patient context and clinical judgment. GD, Gaucher disease; ERT, Enzyme Replacement Therapy; AE, Adverse Event.

## Data Availability

The original contributions presented in the study are included in the article/supplementary material, further inquiries can be directed to the corresponding authors.
