# Supplementary material for: Adverse events signals of enzyme replacement drugs of Gaucher disease: insights from FAERS database analysis
Source: Front Med (Lausanne). 2026 Apr 2;13:1726282. doi: 10.3389/fmed.2026.1726282 (PMC13083113; doi:10.3389/fmed.2026.1726282)
Supplement: Supplementary file 1 [file Table_1.DOCX]

Computational code：

library(readxl)

library(data.table)

ok2 <- data.table(read_xlsx("TTTT.xlsx"))

ok2[,ROR:=((a*d)/(b*c))]

ok2[,RORL:=exp(log(ROR)-1.96*sqrt(1/a+1/b+1/c+1/d))]

ok2[,RORU:=exp(log(ROR)+1.96*sqrt(1/a+1/b+1/c+1/d))]

ok2[,PRR:=(a/(a+b))/(c/(c+d))]

ok2[,XX:=((a*d-b*c)*(a*d-b*c)*(a+b+c+d))/((a+b)*(c+d)*(a+c)*(b+d))]

ok2[,EBGM:=(a*(a+b+c+d))/((a+b)*(a+c))]

ok2[,EBGM05:=exp(log(EBGM)-1.64*(sqrt(1/a+1/b+1/c+1/d)))]

ok2[,IC2:=log2((a*(a+b+c+d))/((a+c)*(a+b)))]

ok2[,GMAE:=((a+b+c+d+2)*(a+b+c+d+2))/((a+b+1)*(a+c+1))]

ok2[,EIC:=log2(((a+1)*(a+b+c+d+2)*(a+b+c+d+2))/((a+b+c+d+GMAE)*(a+b+1)*(a+c+1)))]

ok2[,VIC:=(1/(log(2)))*(1/(log(2)))*((a+b+c+d-3+GMAE)/(3*(1+a+b+c+d+GMAE))+(a+b+c+d-a-b+1)/((a+b+1)*(1+a+b+c+d+2))+(a+b+c+d-a-c+1)/((a+c+1)*(a+b+c+d+3)))]

ok2[,SD:=sqrt(VIC)]

ok2[,BCPNN250:=EIC-2*SD]

ok2[,C025:=IC2-2*SD]

setDT(ok2)

apply(ok2[,.(a,b,c,d)],1,function(x)

{

matrix(x,nrow=2)->inter

chisq.test(inter)$p.value

})->pvalues

ok2[,pvalue:=pvalues]
